# Distinguishing *Kingella kingae* from Pyogenic Acute Septic Arthritis in Young Portuguese Children

**DOI:** 10.3390/microorganisms10061233

**Published:** 2022-06-16

**Authors:** Catarina Gouveia, Ana Subtil, Susana Norte, Joana Arcangelo, Madalena Almeida Santos, Rita Corte-Real, Maria João Simões, Helena Canhão, Delfin Tavares

**Affiliations:** 1 Infectious Diseases Unit, Hospital de Dona Estefânia, Centro Hospitalar Universitário Lisboa Central, 1169-045 Lisbon, Portugal; 2 Nova Medical School, Faculdade de Ciências Médicas, 1169-056 Lisbon, Portugal; helenacanhao@gmail.com; 3 NOVA National School of Public Health, Comprehensive Health Research Center, CHRC, NOVA University Lisbon, 1600-560 Lisbon, Portugal; ana.subtil@ensp.unl.pt; 4 CEMAT, Instituto Superior Técnico, Universidade de Lisboa, 1049-001 Lisbon, Portugal; 5 Pediatric Orthopedic Unit, Área de Pediatria, Hospital de Dona Estefânia, Centro Hospitalar Universitário Lisboa Central, 1169-050 Lisbon, Portugal; snramos22@yahoo.com.br (S.N.); joana.arcangelo@gmail.com (J.A.); delfintavares@gmail.com (D.T.); 6 Laboratory of Molecular Biology, Department of Clinical Pathology, Centro Hospitalar Universitário Lisboa Central, 1169-050 Lisbon, Portugal; maria.santos22@chlc.min-saude.pt (M.A.S.); cortereal.rita@gmail.com (R.C.-R.); 7 Department of Infectious Diseases, National Institute of Health Dr. Ricardo Jorge, 1600-609 Lisbon, Portugal; m.joao.simoes@insa.min-saude.pt

**Keywords:** *Kingella kingae*, acute septic arthritis, pyogenic infections

## Abstract

(1) **Background**: We aim to identify clinical and laboratorial parameters to distinguish *Kingella kingae* from pyogenic septic arthritis (SA). (2) **Methods**: A longitudinal, observational, single-centre study of children < 5 years old with microbiological positive SA admitted to a paediatric hospital from 2013–2020 was performed. Clinical and laboratorial data at admission and at 48 h, as well as on treatment and evolution, were obtained. (3) **Results**: We found a total of 75 children, 44 with *K. kingae* and 31 with pyogenic infections (mostly MSSA, *S. pneumoniae* and *S. pyogenes)*. *K. kingae* affected younger children with low or absent fever, low inflammatory markers and a favourable prognosis. In the univariate analyses, fever, septic look, CRP and ESR at admission and CRP at 48 h were significantly lower in *K. kingae* SA. In the multivariate analyses, age > 6 months ≤ 2 years, apyrexy and CRP ≤ 100 mg/L were significative, with an overall predictive positive value of 86.5%, and 88.4% for *K. kingae*. For this model, ROC curves were capable of differentiating (AUC 0.861, 95% CI 0.767–0.955) *K. kingae* SA from typical pathogens. (4) **Conclusions**: Age > 6 months ≤ 2 years, apyrexy and PCR ≤ 100 mg/L were the main predictive factors to distinguish *K. kingae* from pyogenic SA < 5 years. These data need to be validated in a larger study.

## 1. Introduction

*Kingella kingae*, a Gram-negative aerobic coccobacillus, was first described in the 1960s by Elizabeth King [1]. Some strains can cause invasive disease, and in recent years *K. kingae* has emerged as an important cause of septic arthritis (SA) in children younger than 4 years, ranging from 30% to 93.8% of cases [2,3,4,5,6], mostly due to improved culture techniques and molecular detection methods [7].

*K. kingae* infections usually affect children between 6 to 48 months, are usually milder, have a different microbiological and clinical profile, and require a less aggressive management [1,7,8,9]. In contrast, SA due to pyogenic infections, such as *Staphylococcus aureus* or *Streptococcus* spp., or less commonly due to Gram-negative enteric microorganisms, as occurs more often in older children and adults, is usually associated with high fever and inflammatory parameters, longer days of intravenous (IV) treatment and length of stay (LOS), and has a worse prognosis [2,9,10]. Joint drainage and irrigation are still the standard of care in major joint infections [10,11]. However, the antibiotic choice is a matter of discussion. In children younger than 4 years, a first- or second-generation cephalosporin is recommended to cover for *K. kingae* and also *S. aureus*, as oxacillin does not provide adequate coverage against *K. kingae*. In older children, oxacillin is a good option in countries with a low prevalence of methicillin-resistant *S. aureus* (MRSA), such as Portugal [10,11,12,13,14]. It is thus important to early distinguish *K. kingae* from other SA infections, to guide management and initial antibiotic therapy [2].

Several studies compare the clinical and laboratorial characteristics between *K. kingae* and pyogenic infections, and although differences were noted, few have suggested initial distinguishing features [8,15]. Ceroni et al. [15], in a retrospective study, proposed that fever, CRP above 55 mg/L, leucocytosis above 14,000 cells/mm^3^ and neutrophil band shift ≥ 150 cells/mm^3^ were able to distinguish *K. kingae* from other SA infections. However, this model is still a matter of controversy, lacking validation by other authors [16].

The aim of this study was to compare the signs and symptoms of SA in children under 5 years old caused by *K. kingae* and typical pathogens and to establish predictive parameters to allow the differential diagnosis.

## 2. Materials and Methods

We reviewed all children aged less than 5 years old with microbiological positive SA admitted to a Lisbon paediatric hospital. Clinical, microbiological and imaging data, treatment, complications and sequelae were collected from January 2003 to December 2020. Children less than 3 months of age or that developed infection after surgery, orthopaedic hardware implementation or open trauma were excluded.

Acute SA was defined based on clinical complaints (local pain, swelling, decreased range of movement and imaging findings suggestive of infection) when the duration of symptoms was ≤ 14 days. Only children with a positive culture or molecular bacterial identification were included in this study [10]. Complications and sequelae were considered as previously defined [6].

Microorganism identification and antimicrobial susceptibility were determined by the local microbiology laboratory. Real-time PCR for *K. kingae* was implemented in 2014, using a primer that targeted the *rtxA* gene [17]. Children with arthritis due to *K. kingae* and typical pathogens were compared by bivariate analysis. A multivariate logistic regression was performed to determine which variables at admission best predicted *K. kingae* and pyogenic infections. *K. kingae* diagnostic accuracy was analysed by the area under the ROC curve (AUC) and a cut-off AUC > 0.8 was considered discriminative. All analyses were performed in SPSS Statistics^®^ version 27 (IBM Corp, New York, NY, USA). The study was subject to approval by our Hospital Ethics Committee (EC70-2011, 23 April 2011).

## 3. Results

Of the 75 children with a pathogen-positive SA, the median age was 16.8 [IQR 12–24] months and only 11 were above 36 months: 2 had a chronic disease (one cardiopathy, one cured Wilms tumour), 44 a predisposing factor (28 a previously respiratory infection, 7 trauma, 3 wound and 6 chickenpox), 44 had *K. kingae* and 31 had classical pathogen infections, which included MSSA *(11)*, *S. pneumoniae (7)*, *S. pyogenes (8)*, *H. influenzae type b (2)*, *N. meningitidis (2)* and *Enterobacter aerogenes (1)*. None had co-infections. Clinical and biological features are represented in Table 1.

### 3.1. Kingella kangas

A total of 44 cases of *K. kingae* SA were identified (Table 1), all in children above 6 months and under 4 years, and only 3 (6.8%) were older than 36 months. There were three to eight cases per year, with 59.1% between November and February. Most children (61.4%) reported a preceding respiratory tract infection, suggestive of hand, foot and mouth disease in seven (15.9%), and only 6.8% described previous trauma. Two children were from the same kindergarten, but no additional investigation was undertaken. None had chronic disease.

The most frequent findings of *K. kingae* SA were pain (86.4%), functional limitation (95.5%) and local inflammatory signs (86.4%), but only 16 (37.2%) had fever (tympanic ≥ 38.2 °C). No children looked unwell, but three (6.8%) were described as irritable. The most common affected joints were the knee (38.6%), hip (20.5%) and ankle (18.2%). Most patients had an erythrocyte sedimentation rate (ESR) above 20 mm/h (88.6%) and a peak C-reactive protein (CRP) below 80 mg/L (79. 5%) but below 20 mg/L in only 22.6%. *K. kingae* was identified by molecular amplification in 35 cases (23/27 from synovial fluid and 27/30 from oropharyngeal swab (OPS), only OPS in 8 cases), synovial fluid culture in 14/39 and blood culture in 1/43.

Most patients with *K. kingae* were empirically treated with cefuroxime (93.3%) and were submitted to surgery (86%), mainly due to joint aspiration with lavage (81,8%). Six (18.1%) had arthrotomy and seven (15.9%) had more than one intervention. Complications (11.4%) included intra-osseous abscesses (1), myositis (3) and sub-luxation (1). No patient needed intensive care unit (ICU) admission. At discharge, 10 (22.7%) had symptoms, mostly minor ROM limitation and inflammatory signs. One patient was lost to follow-up. At six months’ follow-up, no patients had sequelae.

### 3.2. Comparing Data between K. kingae and Pyogenic SA

Comparing data between *K. kingae* and pyogenic SA on bivariate analyses, fever at admission, fever duration, fever at 48 h and septic look, CRP and ESR were significantly lower in *K. kingae* infections (Table 1). In addition, the LOS and duration of treatment were also inferior in the *K. kingae* group.

**Figure 1:**
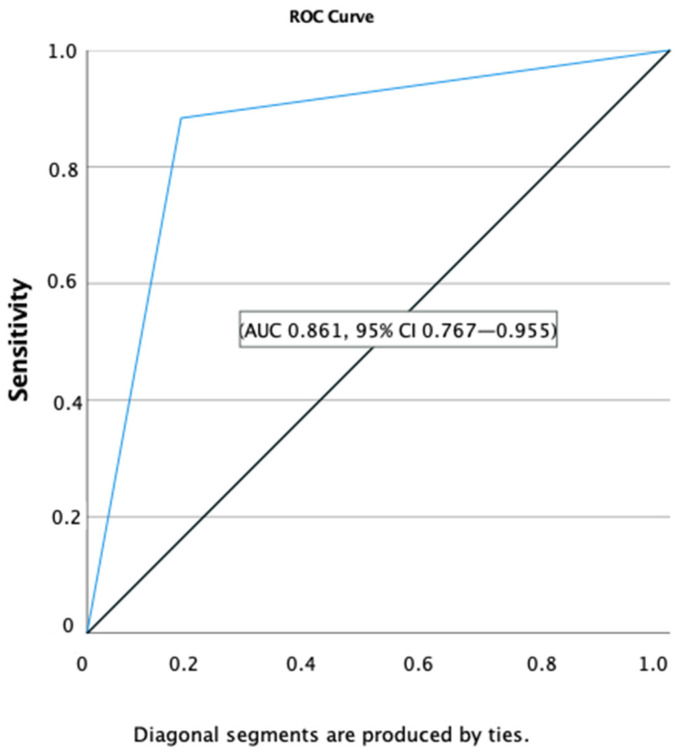
AUC assessed by ROC curves was capable to differentiate *K. kingae* arthritis from typical pathogens, for this model.

We observed that only 37.2% of children with *K. kingae* SA had fever, CRP was higher than 55 mg/L in 34.1% of cases and WBC was elevated in 40%. In comparison, 83.9% of cases with SA due to typical pathogens had fever, CRP level was above 55 mg/L in 80.6% and WBC was elevated in 54.8% of cases. In our cohort, the predictive value for *K. kingae* accounting for WBC count < 14,000 cells/mm^3^ was 63.2% and for CRP < 55 mg/L 82.9%. Applying the Ceroni Score to our cohort, the sensibility (less than two criteria) for *K. kingae* was low (65%), with a good positive predictive value of 86.7%, and for typical pathogens (considering ≥ 2 criteria, as band test was not performed) a better sensibility (87.1%) but a low PPV (65.9%). On multivariate analyses, our best model to distinguish *K. kingae* from pyogenic infections at admission was age above 6 months and ≤2 years, apyrexia and CRP < 100 mg/L, with an overall PPV of 86.5%, 88.4% for *K. kingae* and 83.9% for pyogenic infections (Table 2). For this model, AUC assessed by ROC curves was capable of differentiating (AUC 0.861, 95% CI 0.767–0.955) *K. kingae* arthritis from typical pathogens (Figure 1).

## 4. Discussion

*K. kingae* has recently been recognised as the most important cause of osteoarticular infections in young infants. Furthermore, in Switzerland, France and Spain, *K. kingae* has been reported as the leading cause of OAI in all age groups in children and adolescents [5,7,9,18]. In a previous study of SA infections at our institution (2003–2018), which included 28 of our 44 SA strains, *K. kingae* was already the most frequent bacteria (51.9%) [6].

Although in our series diagnosis was only presumed by oropharyngeal *K. kingae* positivity in eight (18.2%) cases, the predictive positive value of the OPS in young children with osteoarticular infections is very high (91%), making this diagnosis highly probable [19,20]. Furthermore, genotyping of oropharyngeal *K. kingae* without prior culture indicates that these strains matched the most frequent invasive strains [21].

In our study, several common *K. kingae* characteristics can be identified, such as early childhood affection (average 15.3 months, with only 6.8% children older than 36 months), previous upper respiratory symptoms, fall predominance, clinical paucity, low or absent fever, low inflammatory markers and a favourable prognosis [1,3]. All were milder and monoarticular, affecting more commonly the knee, hip and ankle. This low virulence has been evidenced by shorter length of stay, fewer adverse events and a better outcome than pyogenic infections [2,7,15,16], as documented in our series. The number of surgical interventions was similar in both groups, but *K. kingae* infections had shorter duration of treatment and LOS and no admissions to the ICU, confirming the milder nature of these infections. However, 15.9% needed a second intervention, due to persistent effusion, and complications were reported in 11.4%. This is different than that reported by Basmaci et al. [8], with only 1/64 (1,6%) complicated infections that needed a second surgical drainage.

Basmaci and Ceroni have previously characterised the minor increase in biological markers in *K. kingae* arthritis [15,16]. In our study, a lower CRP and ESR were also suggestive of *K. kingae*, [15,22] being less than 100 mg/L in about 88.6% of cases in our cohort, although it could not eliminate classic pathogens, as already suggested [16]. The duration of fever is longer in classic, pyogenic pathogens, with about a third still febrile for more than 48 h after treatment [8,16,23], as observed in our study.

Leukocytes are usually lower than 16,000 cells/mm^3^ [22,24], which is comparable to our patients (12,700 cells/mm^3^), but slightly higher than that reported by Ceroni (10,538 cells/mm^3^) [15]. We did not find any significative difference in leukocyte count between *K. kingae* and classic pathogens, similar to Basmati et al. [16] but different from Ceroni [15].

Ceroni et al. [15] proposed a model to discriminate *K. kingae* from pyogenic osteoarticular infections in children less than 4 years. According to this model, the best predictors of *K. kingae* osteoarticular infections consists of the following four variables: temperature at admission <38 °C, CRP < 55 mg/L, WBC count < 14,000cells/mm^3^ and neutrophil band shift < 159 forms/mm^3^. In our cohort, applying these parameters (with the exception of band shift that we could not quantify), the predictive value for WBC count < 14,000 cells/mm^3^ (63.2%) and CRP < 55 (82.8%) was lower than that reported by Ceroni (77.1% and 90.3%, respectively) [15], but similar to Basmaci [16]. Applying the Ceroni Score to our cohort, the sensibility (less than two criteria) for *K. kingae* was low (65.9%), with a higher positive predictive value (PPV) of 86.7%, and for typical pathogens (considering ≥ two criteria, as band test was not performed) a sensibility of 87.1%, but a low PPV (65.9%). Indeed, these recommended algorithms to differentiate *K. kingae* infections from other pyogenic bacteria have different discrimination power, due to diverse geographic patterns [16] and different applications (Ceroni applied to all osteoarticular infections, Basmati included older children), and cannot be applied to our cohort. Our study provides evidence that age ≥ 6 months but ≤2 years, apyrexy and CRP ≤ 100 mg/L was a better model to distinguish *K. kingae* SA from classic pathogens in children <5 years, with an overall PPV of 86.7%, 88.6% for *K. kingae* and 83.9% for pyogenic infections.

Our study has limitations, such as being a small, single-centre study needing validation in a larger prospective multi-centre study. We plan to increase our sample in the future in order to strengthen the analyses and if possible, include other centres in the study. Furthermore, other parameters such as respiratory rate, heart rate or blood pressure at admission or disseminated disease, being used in severity scores for acute osteomyelitis in children [24,25,26] were not tested and could have improved our analyses.

*K. kingae* SA is less severe than pyogenic SA and needs less aggressive management and a different antibiotic empiric treatment approach. In children with SA under 5 years of age, we propose that the presence of three positive criteria, age ≥ 6 months but ≤2 years, apyrexy and CRP ≤ 100 mg/L, could be reliably used to diagnose *K. kingae* SA at admission.

## Figures and Tables

**Table 1:** *K. kingae* and typical pathogen arthritis in children < 5 years.

	TOTAL N = 75	*K. kingae* N = 44	Typical Pathogens N = 31	*p*
Age, months, median (IQR)	16.8 (12–24)	15.3 (12–24)	18 (9.6–36)	0.623
Age < 36 months, n (%)	64 (85.3)	41 (93.2)	23 (74.2)	0.043
Male gender, n (%)	52 (69.3)	32 (72.7)	20 (64.5)	0.448
Symptom duration at admission, days, median (IQR)	3 (2–5)	3 (2–5)	2 (1–6)	0.220
Fever duration, n (%)	0.5 (0–2)	0 (0–2)	2 (0.75–7.5)	<0.001
Fever at admission, n (%)	42/74 (56.8)	16/43 (37.2)	26 (83.9)	<0.001
Fever > 48 h of antibiotics, n (%)	7/64 (10.9)	0 (0)	7/23 (30.4)	<0.001
Septic look, n (%)	6/71(8.4)	0 (0)	6/28 (21.4)	0.003
Osteoarthritis, n (%)	10 (13.3)	4 (9.1)	6 (19.4)	0.3
Disseminated infection, n (%)	3 (4)	0 (0)	3 (9.6)	0.067
Abscesses, n (%)	3 (4)	1 (2.3)	2 (6.5)	0.566
Myositis, n (%)	10/73 (13.7)	4/43 (9.3)	6/30 (20)	0.3
WBC count, cells/mm^3^, median (IQR)	13,900 (10,800–18,200)	12,700 (10,300–17,100)	15,200 (11,300–19,700)	0.58
WBC < 14,000/mm^3^, n (%)	38/71 (53.5)	24/40 (60)	14 (41.9)	0.214
Platelet’s count, cells/mm^3^, median (IQR)	505,000 (363,000–571,100)	474,500 (376,000–530,500)	554,000 (346,000 690,000)	0.133
CRP peak, mg/L, median (IQR)	61.6 (30–147)	40.5 (18–69)	162 (93.7–215)	<0.001
CRP < 100, mg/L, n (%)	47 (62.7)	39 (88.6)	8 (25.8)	<0.001
CRP at 48–96 h, median (IQR)	27.4 (9.7–79)	16.3 (5–29)	73.3 (30–150)	<0.001
ESR peak, mm/h, median (IQR)	61 (42–79)	54 (39–68.5)	68 (59–94)	0.003
Admitted to ICU, (%)	3 (4)	0 (0)	3 (9.7)	0.067
≥2 surgeries, n (%)	16 (21.3)	7 (15.9)	9 (29)	0.172
Days of IV antibiotic, median (IQR)	10 (5–15)	6 (4–10)	16(13–27)	<0.001
Days of total antibiotic, median (IQR)	25 (21–32.5)	21 (21–26)	32 (26–44)	<0.001
LOS, days, median (IQR)	10 (5–16)	6 (4–11)	16 (11–23)	<0.001
Complications, n (%)	15 (20)	5 (11.4)	10 (32.3)	0.026
Sequelae at 6 months, n (%)	3/73(4.1)	0/43	3/30 (10)	0.065

**Table 2:** Predicted model to distinguish *K. kingae* and typical arthritis pathogens in children < 5 years *.

		Predicted *K. kingae*		
Observed		No	yes	% Correct
*K. kingae*	No	26	5	83.9
	Yes	5	38	88.4
				86.5

* Based on age > 6 months ≤ 2 years, apyrexia and CRP < 100 mg/L.

## Data Availability

The study involved the use of personal, anonymized health data from computerized database in SPSS. The pseudonymization of the data was performed. Gouveia, C.; Duarte, M.; Norte, S.; Arcangelo, J.; Pinto, M.; Correia, C.; Simões, M.J.; Canhão, H.; Tavares, D. *Kingella kingae* Displaced S. Aureus as the Most Common Cause of Acute Septic Arthritis in Children of All Ages. *Pediatr. Infect. Dis. J.* **2021**, *40*, 623–627. https://doi.org/10.1097/inf.0000000000003105 and Gouveia, C.; Branco, J.; Norte, S.; Arcangelo, J.; Alves, P.; Pinto, M.; Tavares, D. Acute haematogenous osteomyelitis in Lisbon: An unexpectedly high association with myositis and arthritis. *An. Pediatr. (Engl. Ed.)* **2022**, *96*, 106–114. https://doi.org/10.1016/j.anpede.2020.11.003, partially used the same database.
